# A Versatile Axle for the Construction of Disassemblage Rotaxanes

**DOI:** 10.3390/molecules21081043

**Published:** 2016-08-10

**Authors:** Lucas A. Powers, David B. Smithrud

**Affiliations:** Department of Chemistry, University of Cincinnati, Cincinnati, OH 45221, USA; powersla@mail.uc.edu

**Keywords:** rotaxanes, disassemble, switch, pH

## Abstract

Rotaxanes are unique mechanical devices that hold great promise as sensors. We report on two new rotaxanes that contain an acid or base sensitive trigger and readily disassemble in a wide range of environments. Disassemblage was observed under TLC and ^1^H-NMR analysis. The axle is highly charged, which enhances solubility in aqueous environments, and can be readily derivatized with sensor components. The trigger was swapped in a one-pot method, which is promising for the rapid production of a series of sensors.

## 1. Introduction

The detection of specific molecules or processes on the molecular scale has greatly benefited a wide range of fields from medicine to environmental protection. This has spurred the rapid growth of chemosensors [1,2]. Chemosensors undergo a physical or chemical change upon interaction with a targeted molecule or environment to produce a signal that is observable to the eye or via instrumental analysis. There are many challenges in creating such sensors. They must reach their target, which requires sufficient stability and solubility, contain an efficient trigger mechanism, and produce a signal intense enough to be sensed. If unique sensors had to be designed and built for every target, this would be a daunting challenge.

The rotaxane architecture provides a promising universal framework for sensor development [3,4,5,6,7]. [2]Rotaxanes comprise a circular molecule (wheel) threaded over a linearly shaped molecule (axle) with large groups (blocking groups) attached to the axle’s ends to keep the wheel threaded. Most of the original rotaxane-sensors operated by the wheel switching positions on the axle after an external stimulus is applied to give an observable signal [8,9,10,11,12,13]. While this class of sensors continues to show great promise, we are constructing sensors that operate via the controlled disassemblage of a rotaxane. Literature results show that an external stimulus can reduce the stability of pseudorotaxane (a rotaxane with one blocking group)/rotaxanes, promoting disassemblage. For example, Martinez-Cuezva et al promoted the dethreading of a [2]rotaxane using heat, flash vacuum pyrolysis, or microwave radiation [14]. Leung showed that reducing the interaction strength between axle and wheel via deprotonation or switch of solvent accelerated disassemblage [15]. Even the simple protonation-deprotonation of a blocking group can provide control of rotaxane formation [16]. Recently, Huang showed that a polyrotaxane can be reversibly assembled and disassembled using irradiation and heating steps [17].

Our goal is to construct rotaxane-sensors that disassemble when an external agent reduces the size of a blocking group and operate in biological solutions. The first step is to develop an axle that can be readily derivatized with sensor components. The axle cannot impede the release of the wheel and be highly soluble in water. Finally, the wheel needs to efficiently thread onto an axle for high yields of a pseudorotaxane prior to rotaxane synthesis using the end-capping method [18]. Thus, a potential axle needs to display the necessary charge state and solubility to enable wheel threading for rotaxane formation while still being water soluble for sensor operation. Herein, we report on an optimal axle that contains two alkylammonium ions for enhanced water solubility, forms pseudorotaxanes in good yields, enabling rotaxane formation, and promotes rapid disassemblage once the blocking group is reduced in size.

## 2. Results and Discussions

### 2.1. Synthesis of the [2]Rotaxanes

**Figure 1:**
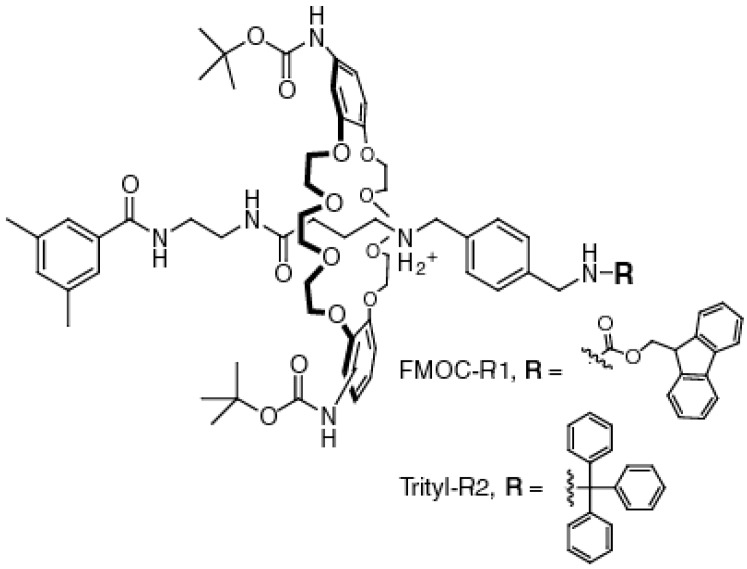
FMOC-R**1** and Trityl-R**2** were constructed and investigated as disassemblage rotaxanes.

[2]Rotaxanes FMOC-R**1** and Trityl-R**2** were constructed (Figure 1) to develop the axle, find a route for rotaxane synthesis, and test the switches. Since many biological environments display unique pH values, such as the acidic environment of cancerous sites [19,20,21], changes in pH were used to reduce the size of the blocking group. Amine bases found in the body, such as serotonin, are attractive targets for monitoring health [22,23]. The FMOC group of FMOC-R**1** is cleaved under basic conditions, whereas, the trityl group of Trityl-R**2** is cleaved under acidic conditions. A Boc-protected, dibenzyl-24-crown-8 ether was used as the wheel, and a 1,3-dimethyl benzenoid ring was used as a model blocking group. Future [2]rotaxanes will contain various signal groups along with appropriate triggers, depending on the need of a sensor. Biological solutions also have a variety of polarities, therefore we measured disassemblage in chloroform, DMSO, and aqueous solutions.

**Scheme 1:**
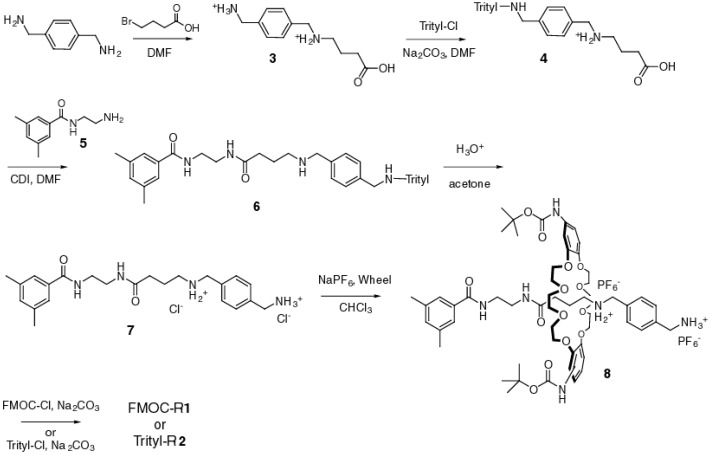
Synthesis of the [2]rotaxanes.

The [2]rotaxanes were readily synthesized in a few steps in an overall 20% yield (Scheme 1). The short, highly polar axle **3** was chosen to enhance the solubility of the rotaxanes in aqueous solutions with an eye towards constructing biologically active sensors. Having an amino and carboxy termini enables a wide range of signal and trigger groups to be coupled either through nucleophilic or electrophilic atoms. Axle **3** was synthesized through amide coupling of 4-bromobutyric acid with 1,4-xylene diamine. It was purified by simple extraction into water, which demonstrates its high polarity, followed by recrystallization. After a protecting group was added to its benzylic amine (we have used FMOC, BOC, and trityl), the carboxylic acid was activated with CDI, followed by the addition of blocking group **5**. Once the protecting group was removed, the alkylammonium ions were converted to PF_6_^−^ salts to promote pseudorotaxane **8** formation. Blocking group-axle **7**, as the PF_6_^−^ salt, and wheel were combined in a minimal amount of chloroform, followed by the addition of Trityl-Cl or FMOC-Cl. The [2]rotaxanes were obtained in good yields of 50% after purification via column chromatography. The wheel and blocking group-axle **7** were also recovered and reused.

Adding the trigger in the last step is convenient for synthesizing a set of sensors for different targets. The trigger can be switched readily in a one-pot method. For example, FMOC-R**1** was readily converted to Trityl-R**2**. FMOC-R**1** was exposed to piperidine in a minimal amount of DMSO. After removing DMSO via evaporation, a minimal amount of dried chloroform was added to the pseudorotaxane **8** followed by the addition of Trityl-Cl. Trityl-R**2** was produced in a non-optimized, 30% yield.

### 2.2. Measuring the Stability of Pseudorotaxane ***8***

**Figure 2:**
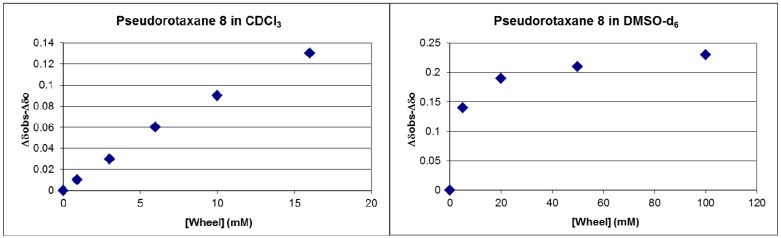
Changes in the chemical shift of an amide proton of pseudorotaxane **8** held at a constant concentration in CDCl_3_ and DMSO-*d*_6_ (10 mM and 30 mM, respectively) caused by the increasing concentration of the wheel.

To operate as a sensor, the pseudorotaxane needs to disassemble once the trigger is activated. The stability of pseudorotaxane **8** was determined in chloroform, DMSO, and a DMSO/water mixture (Figure 2). These solvents were chosen to represent environments a sensor may operate in in a biological system. Furthermore, blocked-axle **7** was investigated as the chloride salt, which will likely exist in biological environments. In CDCl_3_, the strong ion pairs greatly weakens pseudorotaxane **8** (*K*_A_ = 17 ± 0.6 M^−1^) when compared to the typical association observed in the millimolar range for the complex of DB24C8 with R_2_NH_2_^+^ PF_6_^−^ salts [24,25,26]. Tighter association was observed in DMSO-*d*_6_ (*K*_A_ = 250 ± 10 M^−1^). This value is consistent with literature values [27,28,29]. Association was not observed in a 70/30 (*v*/*v*) mixture of DMSO-*d*_6_/D_2_O up to a 50 mM solution of both components. This finding is not surprising considering that water forms strong hydrogen bonds that out-compete the favorable charge-induced dipole interactions between the alkylammonium ions of blocked-axle **7** and the wheel. These results show that the pseudorotaxanes will disassemble in the high millimolar concentration range for most environments in biological systems. For future sensors, additional potential favorable interactions between the wheel and sensor components would have to be considered.

### 2.3. Investigating the Rate of Disassemblage

Sensors need to disassemble quickly after a trigger is activated to localize the target of interest. FMOC-R**1** and Trityl-R**2** were tested for the rate of wheel slippage. Thin layer chromatography analysis was used initially. The three components of FMOC-R**1** and Trityl-R**2** (wheel, axle, and the FMOC or trityl byproducts) are clearly differentiated from the starting rotaxanes via TLC. The rate of trityl clippage was sluggish, requiring a very high percentage of acid to be observed within 30 min (for example, 50% TFA in CHCl_3_). On the other hand, exposing FMOC-R**1** to ethanolamine or piperidine from a 5% (*v*/*v*) solution down to 2 equivalence of amine to rotaxane in DMSO resulted in rapid disassemblage of the rotaxane within two minutes. FMOC cleavage via amines is sluggish in chloroform so *t*-butoxide dissolved in a minimal amount of DMSO (5% *v*/*v*) was used instead. Once again, FMOC-R**1** disassembled within five minutes after exposing it to solutions containing 2 eq. of *t*-butoxide to FMOC-R**1** in CHCl_3_.

**Figure 3:**
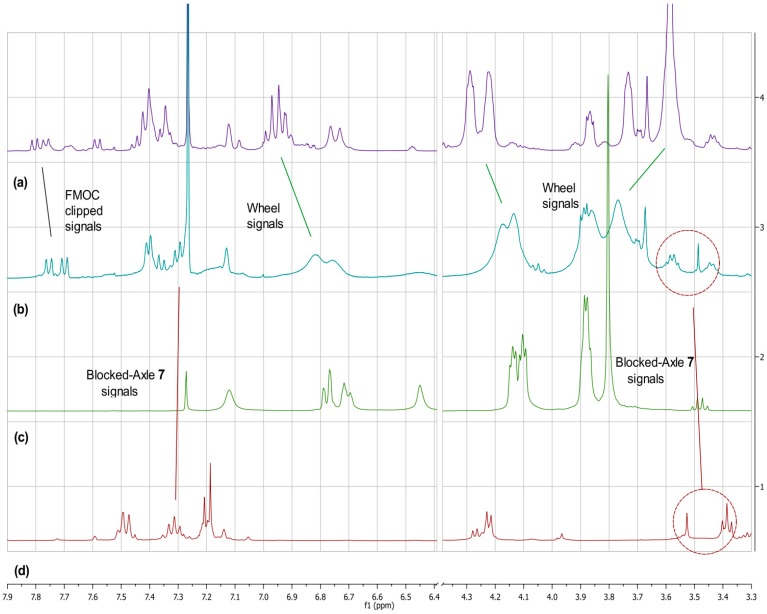
Example of the disassemblage of FMOC-R**1** caused by the presence of piperidine. (**a**) FMOC-R**1** (2 mM) in CDCl_3_/DMSO-*d*_6_ (95%/5% *v*/*v*); (**b**) the same solution as (**a**) with the addition of piperidine (1% *v*/*v*) after approximately 5 min. Disassemblage is evident with the chemical shifts of the wheel and blocked-axle **7** more closely matching the chemical shifts of the pure wheel and blocked-axle **7** (**c**,**d**), respectively.

^1^H-NMR analysis confirmed rapid disassemblage. We needed to demonstrate that pseudorotaxane **8** disassembled quickly. FMOC-R**1** was chosen since the FMOC group is rapidly removed in a small percentage of base. For these studies, the concentration of FMOC-R**1** was kept low (2 mM) to ensure that only an insignificant amount of pseudorotaxane **8** would exist after FMOC clippage. Under all conditions (2 eq. t-butoxide or 1% piperidine in 95/5 CDCl_3_/DMSO-*d*_6_, 1% ethanolamine in DMSO-*d*_6_, and 1% ethanolamine in 70/30 DMSO-*d*_6_/D_2_O), FMOC-R**1** was disassembled within 5 minutes, which is the minimum time needed to run an ^1^H-NMR experiment. Figure 3 highlights the shifts in ^1^H-NMR spectra for FMOC-R**1** in CDCl_3_ caused by the addition of piperidine (see Supplementary Materials for results obtained in DMSO-*d*_6_). These results are consistent with the slippage rates of axles containing benzylammonium ions, which are in the millisecond range [30]. These findings suggest that sensors built from Axle **3** should rapidly disassemble and accurately identify the location of a desired target in a variety of environments, including aqueous environments.

## 3. Materials and Methods

### 3.1. General Synthetic Protocol

Solvents, reagents, and starting materials for synthesis were purchased from Sigma-Aldrich (St. Louis, MO, USA). Moisture sensitive reactions were carried out under positive argon pressure. All organic solvents were freshly distilled over a suitable drying agent. ^1^H- and ^13^C-NMR spectra were obtained using a Bruker AMX400 spectrometer (The Woodlands, TX, USA) operating at 400.13 MHz for proton and 100.61 MHz for carbon nuclei. Chemical shifts are in ppm and referenced using an internal TMS standard for ^1^H-NMR or deuterated solvent for ^13^C-NMR. ESI mass spectra were obtained using Micromass Q-TOF-2 spectrometer. Samples were injected from a MeOH/formic acid solution.

### 3.2. Synthetic Procedures

**Axle 3:** 1,4-Xylene diamine (2.0 g, 15 mmol) was dissolved in 20 mL of dry DMF. Sodium carbonate was added (3.1 g, 29 mmol) to the solution. In a separate flask, 4-bromobutyric acid (2.5 g, 15 mmol) was dissolved in 1 mL of dry DMF and deprotonated with 1.5 equivalences of triethylamine. 4-Bromobutyrate was added dropwise to the xylene diamine solution over 1 hour. The solution was then stirred at 70 °C for 16 hours. Solid Na_2_CO_3_ was removed via filtration, and the solvent was removed under vacuum. The resulting solid was extracted CH_2_Cl_2_/water (3×). The aqueous phase was kept, and water was removed under vacuum. The residue was recrystallized in 10% MeOH/90% CH_2_Cl_2_ (*v*/*v*). Solid 1, 4-Xylene diamine was removed via filtration, leaving axle 3 as an orange oil in a 72% yield (2.35 g, 10.6 mmol). ^1^H-NMR: (D_2_O): δ 1.66 (2H, m), 2.17 (2H, t), 3.38 (2H, t), 4.21–4.28 (4H, m), 7.27 (4H, S). ^13^C-NMR: δ 27.76, 32.32, 38.75, 42.47, 60.76, 127.77, 129.11, 131.54, 133.35, 136.89, 138.98, 176.14. Mass spectral analysis for C_12_H_18_N_2_O_2_-H^+^ (cal. 223.14 found 223.14).

**Trityl-Axle 4:** Axle **3** (2.0 g, 9.0 mmol) was dissolved in 20 mL of dry DMF. Sodium carbonate was added (950 mg, 9.0 mmol) to the solution, followed by the addition of trityl chloride (2.5 g, 9.0 mmol). The solution was stirred at 70 °C for 16 h. The solution was filtered to remove Na_2_CO_3_, and the DMF was removed under vacuum. The crude product was extracted with CH_2_Cl_2_/pH = 7 water (3×). The organic layers were collected and concentrated. The crude product was purified via column chromatography (100% CH_2_Cl_2_, then 5% to 50% methanol). Trityl-Axle **4** was obtained as an orange oil in 80% yield (3.4 g, 7.0 mmol). ^1^H-NMR (DMSO-*d*_6_): δ 1.45 (2H, m), 2.00 (2H, t), 3.22 (2H, t), 3.95–4.05 (4H, m), 6.80–7.00 (16H, m), 7.20 (3H, d). ^13^C-NMR: δ 27.54, 32.12, 38.67, 42.39, 60.83, 80.25, 125.35, 127.23, 127.58, 128.78, 129.21, 129.35, 129.78, 130.56, 131.62, 133.22, 136.95, 139.07, 176.28. Mass spectral analysis for C_31_H_32_N_2_O_2_-H^+^ (cal. 465.25 found 465.20).

**Blocking Group 5:** A solution containing 3,5-dimethyl benzoic acid (2.0 g, 13 mmol) in 20 mL of dry DMF was cooled to 0 °C. CDI was added (2.2 g, 15 mmol) to the reaction mixture, which was stirred under Ar. After two hours, the solution was transferred to a dropping funnel. It was added dropwise over two hours to a solution containing ethylene diamine (1.6 g, 26.6 mmol) in 5 mL of dry DMF. The reaction mixture was stirred at room temperature for 16 h. DMF was evaporated, and the crude product was purified by column chromatography (5% to 20% methanol). Blocking group **5** was obtained as a yellow oil in 85% yield (2.2 g, 11 mmol). ^1^H-NMR (DMSO-*d*_6_): δ 2.30 (6H, s), 2.89 (2H, t), 3.46 (2H, m), 7.08 (1H, s), 7.42 (2H, s). ^13^C-NMR: δ 21.23, 43.51, 61.47, 129.58, 130.32, 130.96, 136.28, 137.41, 138.42, 170.21. Mass spectral analysis for C_11_H_16_N_2_O-Na^+^, (cal. 215.12 found 215.18).

**Blocked-Trityl-Axle 6:** A solution of trityl-axle **4** (1 g, 2.1mmol) in 10 mL of dry DMF was cooled to 0 °C, and CDI (350 mg, 2.3 mmol) was added. After two hours, blocking group **5** (413 mg, 2.1 mmol) was added to the reaction mixture. The reaction mixture was warmed to room temperature and stirred for 16 hours. DMF was removed via evaporation under vacuum, and the crude material was extracted in CH_2_Cl_2_/1N NaOH. The organic layer was concentrated, and the material was purified via column chromatography (100% CH_2_Cl_2_, then 5% to 20% methanol). Blocked-trityl-axle **6** was obtained as a yellow oil in 73% yield (1.0 g, 1.6 mmol). ^1^H-NMR (DMSO-*d*_6_): δ 1.65 (2H, m), 2.33 (6H, s), 2.38 (2H, m), 3.37 (2H, m), 3.49 (2H, m), 3.64 (2H, t), 4.38–4.44 (4H, m), 7.00–7.50 (22H, m). ^13^C-NMR: δ 21.26, 27.59, 32.18, 38.72, 42.35, 43.56, 60.75, 61.50, 80.28, 125.32, 127.31, 127.63, 128,72, 129.26, 129.42, 129.51, 129.82, 130.39, 130.53, 130.91, 131.57, 133.27, 136.25, 136.91, 137.43, 138.45, 139.02, 170.27, 176.31. Mass spectral analysis for C_42_H_46_N_4_O_2_-H^+^, NaCl (cal. 697.33 found 697.30).

**Blocked-Axle 7:** Blocked-trityl-axle **6** (600 mg, 0.94 mmol) was exposed to 200 μL of 1N HCl in 10 mL of methanol. The reaction mixture was stirred at room temperature for 4 hours. Sodium bicarbonate was added to neutralize the solution. Solid Na_2_CO_3_ was removed via filtration, and the methanol was evaporated under vacuum. The crude product was washed with ether (3×) to remove the trityl by-products. Blocked-axle **7** was obtained as a yellow solid in 95% yield (350 mg, 0.89 mmol). ^1^H-NMR: δ 1.68 (2H, m), 2.20 (6H, s), 2.30 (2H, m), 3.15 (2H, m), 3.32 (2H, m), 3.40 (2H, t), 4.22–4.29 (4H, m), 7.14 (1H, s), 7.18–7.23 (4H, m), 7.49 (2H, s). ^13^C-NMR: δ 21.24, 27.53, 32.16, 38.70, 42.31, 43.59, 60.73, 61.55, 125.35, 127.33, 129.47, 129.80, 130.87, 131.61, 133.31, 136.22, 136.94, 137.40, 138.41, 138.96, 170.22, 176.27. Mass spectral analysis for C_23_H_32_N_4_O_2_-H^+^, MeOH (cal. 429.29 found 429.22).

**FMOC-R1:** Blocked-axle **7** (600 mg, 1.5 mmol) was suspended in a diethyl ether/ethyl acetate (50/50 (*v*/*v*)) solution. Sodium hexafluorophosphate (300 mg 1.8 mmol) was added, and the mixture was extracted with pH = 3 water. Organic solvents were evaporated under vacuum, and the residue was dissolved in 0.5 mL of freshly distilled chloroform. The wheel (400 mg, 0.6 mmol) was added, and the solution was stirred under Ar. After 30 min, FMOC-Cl (390 mg, 1.5mmol) was added to the solution. The reaction mixture was stirred at room temperature for 12 h. Excess Na_2_CO_3_ was added to the reaction mixture, which was stirred for an additional 12 h. The solution was filtered to remove Na_2_CO_3_, and CHCl_3_ was evaporated under vacuum. The crude material was purified via column chromatography (100% CH_2_Cl_2_, then 3% to 5% methanol). FMOC-R**1** was isolated as a red foam in a 52% yield (400 mg, 0.3 mmol). ^1^H-NMR (CDCl_3_): δ 1.50 (18H, s), 2.07 (2H, t), 2.27–2.31 (8H, m), 3.41 (2H, t), 3.50–4.38 (35H, m), 6.78–7.80 (21H, m). ^13^C-NMR: δ 21.19, 28.38, 31.46, 35.42, 36.60, 40.81, 41.11, 46.57, 46.92, 52.17, 53.59, 66.74, 67.03, 67.36, 68.05, 69.60, 70.79, 77.36, 80.50, 106.66, 113.11, 113.59, 120.33, 124.91, 127.24, 127.39, 127.76, 128.42, 133.05, 133.93, 134.00, 134.59, 135.02, 138.10, 158.12, 162.74, 168.88, 169.12. Mass spectral analysis for C_72_H_92_N_6_O_16_-H^+^, HCl, HCO_2_H, NaPF_6_ (cal 1547.60 found 1547.64).

**Trityl-R2:** The same procedure was followed for FMOC-R**1** synthesis with Trityl-Cl replacing FMOC-Cl. Trityl-R**2** was isolated as a yellow oil in a 51% yield (400 mg, 0.3 mmol). ^1^H-NMR (CDCl_3_): δ 1.50 (18H, s), 2.27 (2H, t), 2.32 (6H, s), 2.38 (2H, m), 3.41 (2H, m), 3.54 (2H, m), 3.65–4.48 (28H, m), 6.82 (6H, m), 6.96–7.8 (14H, m). ^13^C-NMR: δ 21.16, 28.12, 28.37, 30.93, 33.89, 40.97, 43.41, 47.57, 52.51, 69.03, 69.54, 70.34, 77.31, 80.37, 106.05, 111.88, 115.76, 124.81, 126.42, 127.29, 127.83, 127.91, 128.06, 128.11, 128.19 128.60, 133.10, 134.13, 138.20, 145.96, 149.19, 153.10, 158.15, 168.60. Mass spectral analysis for C_76_H_96_N_6_O_14_-H^+^, MeOH, H_2_O, NaCl (cal 1425.74 found 1425.71).

### 3.3. Measuring the Association Constants

Complex formation between pseudorotaxane **8** and the wheel were investigated in various solutions (CDCl_3_, DMSO-*d*_6_, and 70/30 (*v*/*v*) DMSO-*d*_6_ to D_2_O) at 25.0 °C. For the assay performed in CDCl_3_, a constant concentration of pseudorotaxane **8** (10 mM) in 0.5 mL CDCl_3_ was exposed to an increasing amount of wheel. The wheel was added to the NMR tube via small volume additions from a stock solution of wheel in CDCl_3_ (0.15 M). The change in volume of 11% after the final addition of wheel was not considered in the determination of the association constant. A similar procedure was followed for the assays performed in the DMSO-*d*_6_, except that the concentration of the wheel-stock solution was higher (25 mM). For the assay performed in 70/30 (*v*/*v*) DMSO-*d*_6_ to D_2_O, the wheel and pseudorotaxane **8** were mixed to give a final 50 mM solutions of both components. At a higher concentration, precipitation was observed. To obtain association constants, shifts in the chemical shift of an amide proton of pseudorotaxane **8**, caused by changes in a wheel’s concentration, were plotted and fitted, using non-linear least-square fitting procedure to derive the association constants [31].

The following binding equation was used to calculate the association constants, ∆δ = ∆δ_obs_ − ∆δ_0_ = *K*_A_[T] ∆δ_max_ / (1 + *K*_A_[T]), where the difference in the chemical shift (∆δ) of an axle proton in the presence of wheel (∆δ_obs_) and in its absence (∆δ_0_) depends on the concentration of the wheel (T), the chemical shift of the proton when it is completely bound to the wheel (∆δ_max_), and the association constant of this complex (*K*_A_).

### 3.4. Observing Disassemblage

A series of 1 dram vials containing a dilute solution of a rotaxane (0.5 mM) was exposed to various bases at different concentrations. After addition of a base, the samples were run at various time points on a TLC plate and the amount of rotaxane was estimated via eye. For the ^1^H-NMR assays, a ^1^H-NMR spectrum was taken of a 1 mM solution of FMOC-R**1** in CDCl_3_ or DMSO-*d*_6_ with or without a cosolvent. A base was added to this solution to give a final solution containing: 2 eq. *t*-butoxide or 1% piperidine in 95/5 CDCl_3_/DMSO-*d*_6_, 1% ethanolamine in DMSO-*d*_6_, or 1% ethanolamine in 70/30 DMSO-*d*_6_/D_2_O. A second ^1^H-NMR spectrum was taken immediately after adding a base.

## 4. Conclusions

A new class of disassemblage [2]rotaxanes were successfully constructed and shown to disassemble readily in a variety of solvents once the trigger was activated. The [2]rotaxanes were synthesized in a few steps, resulting in a good, overall yield from starting materials of 20%. The pseudorotaxane is stable enough as the PF_6_^-^ salt to form [2]rotaxanes in good yields, while as with the chloride salt, the wheel is only weakly threaded, resulting in dethreading in the millimolar concentration range in CDCl_3_, DMSO-*d*_6_, and in DMSO-*d*_6_/D_2_O mixtures. Disassemblage is also rapid, which is important for future sensors to locate a target of interest. The axle is very versatile. It can be readily derivatized with sensor components and is highly polar once the trigger is activated. High polarity is crucial for developing sensors that operate in biological solutions. We also demonstrated that the trigger can be swapped efficiently in a one pot, synthetic procedure.

## Supplementary Materials

Supplementary materials can be accessed at: http://www.mdpi.com/1420-3049/21/8/1043/s1.
