# Supplementary material for: A Versatile Axle for the Construction of Disassemblage Rotaxanes
Source: Molecules. 2016 Aug 10;21(8):1043. doi: 10.3390/molecules21081043 (PMC6274001; doi:10.3390/molecules21081043)
Supplement: Supplementary file 1 [file molecules-21-01043-s001.pdf]

Molecules **2016**, *21*, 1043; doi:10.3390/molecules21081043

Supplementary Materials: A Versatile Axle for the Construction of Disassemblage Rotaxanes

Lucas A. Powers and David B. Smithrud

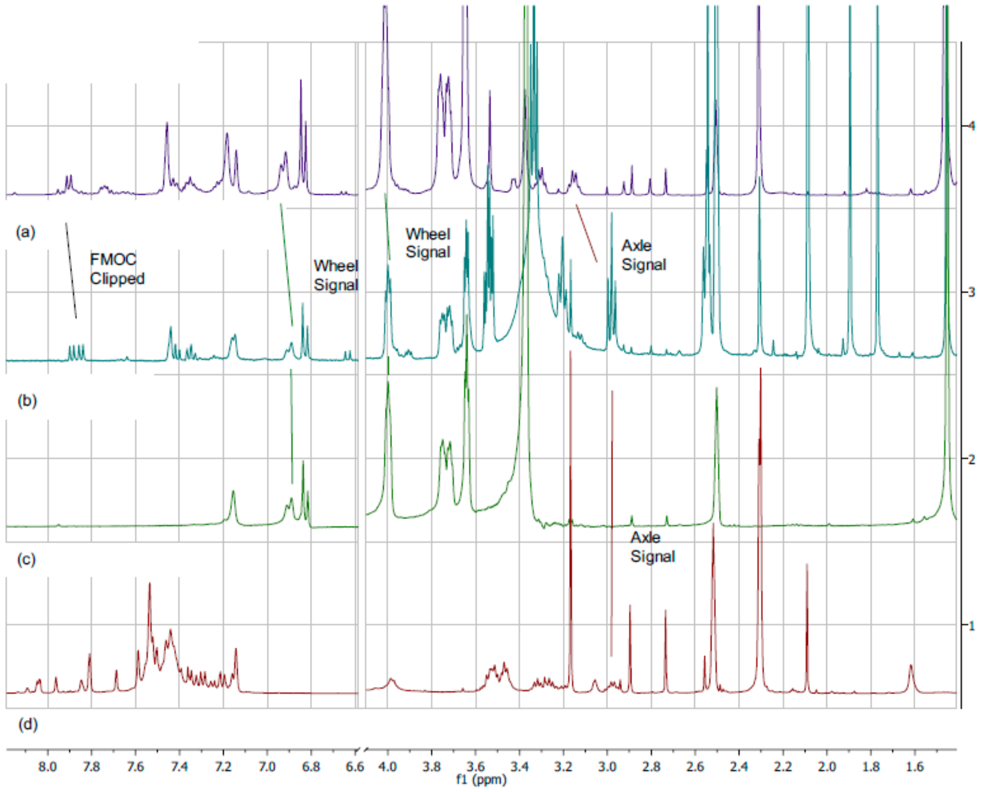

Figure S1. Example of the disassemblage of FMOC-R**1** caused by the presence of piperidine. (**a**) FMOC-R**1** (2 mM) in DMSO-d_6, (**b**) the same solution as (**a**) with the addition of ethanolamine (1% *v*/*v*) after approximately 5 minutes. Disassemblage is evident with the chemical shifts of the wheel and blocked-axle **7** more closely matching the chemical shifts of the pure wheel and blocked-axle **7** (**c**,**d**), respectively.
